# Cost-effectiveness analysis of 23-valent pneumococcal polysaccharide vaccine for adults in China

**DOI:** 10.1016/j.imj.2026.100258

**Published:** 2026-04-27

**Authors:** Yamin Shu, Shuhua Tan, Yao Chen, Yiling Ding, Pingping Xu, Qilin Zhang

**Affiliations:** a Department of Pharmacy, Tongji Hospital, Tongji Medical College, Huazhong University of Science and Technology, Wuhan 430030, China; b International Research Center for Medicinal Administration, Peking University, Beijing 100191, China; c Department of Statistical Sciences, Univerisity of Toronto, Toronto M5S6E8, Canada; d Department of Colorectal Surgery, Zhongshan Hospital, Fudan University, Shanghai 200032, China; e Department of Pharmacy, Union Hospital, Tongji Medical College, Huazhong University of Science and Technology, Wuhan 430022, China

**Keywords:** Cost-effectiveness, 23-valent pneumococcal polysaccharide vaccine, Pneumococcal disease, Vaccine adverse event reporting system, Societal perspective

## Abstract

**Background:**

With population aging and the rising burden of chronic diseases, pneumococcal disease imposes increasing pressure on the healthcare system in China. This study evaluated the cost-effectiveness of the 23-valent pneumococcal polysaccharide vaccine (PPSV23) in adults aged 50 years.

**Methods:**

A decision-tree–based static cohort Markov model was developed to estimate lifetime costs and health outcomes from a societal perspective under a hypothetical strategy of PPSV23 vaccination at age 50 with revaccination at age 65 to assess long-term protection under waning immunity; a single-dose strategy was examined in scenario analyses. The model estimated quality-adjusted life-years, life-years, incremental cost-effectiveness ratios, and cases and deaths averted. Deterministic and probabilistic sensitivity analyses were conducted to assess parameter uncertainty. Price simulations were performed across different willingness-to-pay thresholds. Safety was evaluated using the Vaccine Adverse Event Reporting System through descriptive analysis and empirical Bayesian data mining.

**Results:**

The estimated incremental cost-effectiveness ratios for PPSV23 compared to no vaccination are $12,937.07 per quality-adjusted life-year gained and $9,622.25 per life-year gained, with the administration of PPSV23 preventing 1,115 cases and 35 deaths from a study population of 100,000 individuals. The greatest sensitivity was observed to variations in the discount rate, serotype coverage of PPSV23, vaccine efficacy of PPSV23 and cost per dose of PPSV23. No new or unexpected safety concerns for PPSV23 were identified.

**Conclusions:**

PPSV23 is a cost-effective option for adults aged 50 years in China at higher willingness-to-pay thresholds (e.g., 2–3 × the gross domestic product per capita), with a favorable post-marketing safety profile.

## Highlights

• The 23-valent pneumococcal polysaccharide vaccine (PPSV23) is cost-effective at willingness-to-pay thresholds of 2–3 × the gross domestic product per capita.
• PPSV23 prevents 1,115 cases and 35 deaths from a population of 100,000 individuals.
• Result was most sensitive to variations in the discount rate and serotype coverage.
• No new or unexpected safety concerns for PPSV23 were identified.

## Graphical abstract

**Figure:**
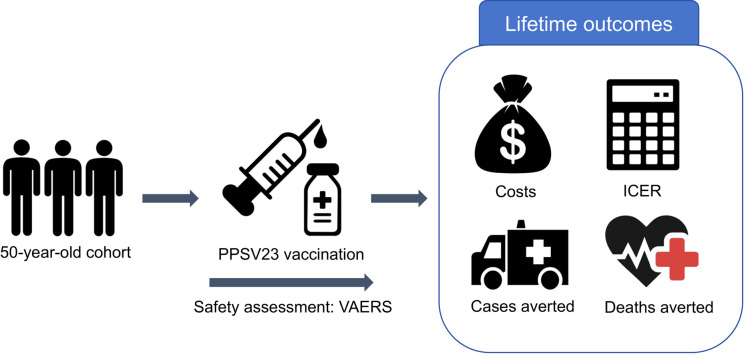

## 1 Introduction

*Streptococcus pneumoniae* (*S. pneumoniae*) is the leading cause of invasive pneumococcal disease (IPD) and community-acquired pneumonia (CAP) and is a significant source of morbidity and mortality in adults aged ≥ 50 years.^1^ About 30%–50% of CAP hospitalizations are associated with *S. pneumoniae* infection globally.^2^ In the United States, the incidence of hospitalization due to CAP is approximately 63.0 per 10,000 person-years in those older than 65 years.^3^ The 30-day mortality rate after CAP hospitalization was 26.8% in patients aged 60 years and older with comorbidities.^3^ Pneumococcal disease (PD), including IPD and CAP, poses a substantial disease burden among young children and older adults in China.^4^^,^^5^ The incidence of CAP in 2018 was estimated at 29.8 to 221.0 cases per 10,000 people, with people aged ≥ 65 years accounting for 37% of all cases.^6^ The WHO has identified PD as a “very high priority” vaccine-preventable disease due to the high disease burden and the emergence of resistance to antimicrobial treatments.^7^

A number of multivalent vaccines, such as the 7, 10, 13, 15, and 20-valent pneumococcal conjugate vaccine (PCV7, PCV10, PCV13, PCV15 and PCV20), and the 23-valent pneumococcal polysaccharide vaccine (PPSV23) have been developed and widely used to prevent PD.^8^^,^^9^ Currently, only PCV13 and PPSV23 are available in China. PCV13 has been used to prevent IPD in children. PPSV23, which can cover about 90% of the pathogenic serotypes was first developed in the United States in 1983, and has shown promising efficacy.^10^ Many developed countries, such as the United States, and United Kingdom, incorporated PPSV23 into their national immunization programs for adults 65 years and older to reduce the medical burden of pneumococcal infections.^11^ China approved the use of PPSV23 vaccine in 1996, which was recommended by the Chinese Center for Disease Control and Prevention for people over 2 years of age at high risk of PD.^12^ Furthermore, PPSV23 is the only pneumococcal vaccine approved for use in the elderly in China.

Although some studies have shown that PPSV23 is cost-effective in people aged ≥ 60 years in Shanghai, China, another economic study confirmed that PCV13 vaccination in Chinese seniors (≥ 65 years) is more cost-effective than PPSV23 vaccination and PCV13-PPSV23 sequential vaccination.^9^^,^^11^^,^^13^ It is unclear whether starting vaccination at an earlier age would be a more efficient use of public resources. Unfortunately, no national guidelines have been developed for PPSV23 currently. According to the vaccine specification, PPSV23 has been designed for vaccination in adults over 50 years of age. However, to the best of our knowledge, no domestic studies have reported the cost-effectiveness of PPSV23 in this population.

Due to the limited evidence and health resources on the value of PPSV23 vaccination in older adults, PPSV23 immunization program has not been considered a priority in China. In addition, considering the recognized limitations of a single clinical trial or case report in assessing the safety profile of PPSV23^14^^,^^15^, a comprehensive overview on the adverse events (AEs) of PPSV23 based on the real-world large-sample data is still lacking, which significantly limits the widespread use of PPSV23. Therefore, an expanded population economic and safety analysis is warranted to evaluate the cost-effectiveness and safety of PPSV23 for PD prevention. The purpose of this study was to determine the cost-effectiveness and potential health benefits of PPSV23 in the prevention of pneumonia and other PD in adults aged 50 years, as well as safety, and to provide possible policy decisions for future expanded use of pneumococcal vaccines in China.

## 2 Materials and methods

### 2.1 Model overview

We employed a decision-tree-based static cohort Markov model from the societal perspectives to estimate the costs and epidemiological impact of various vaccination scenarios, including administering a single dose of PPSV23 to older adults at 50 and revaccination at 65 years old, as well as no vaccination. The model using hypothetical cohorts of 50 years old adults would adopt a lifetime horizon (up to a maximum age of 100 years) with an annual cycle length, considering the potential risk of PD throughout the lifespan of the elderly population. Our model estimated the disease burden, including the number of cases and deaths attributed to IPD and CAP, as well as the associated costs. Assuming all individuals enter the model in a healthy state with an inherent susceptibility to pneumococcal infection, encompassing three disease events (CAP, meningitis, and bacteremia).^1^ According to the severity of the condition, CAP can be categorized into two distinct types: outpatient and inpatient. The model assumes that outpatient patients will not experience fatal outcomes; however, inpatient pneumonia, bacteremia, and meningitis may result in sequelae or mortality. Once patients develop sequelae, they are confined to a state of either permanent disability (including hearing loss, cognitive impairment, motor dysfunction, and epilepsy) or mortality.^11^ In contrast, patients in other states experiencing CAP and IPD can achieve complete recovery. The Markov model culminates in 15 mutually exclusive outcomes after a series of progressive iterations (Supplementary Fig. S1). In order to streamline the model, we excluded AEs and herd immunity due to their limited impact on outcomes and lack of available data.

The costs in the model are discounted using US dollars (1 USD = 7.05 RMB).^16^ Both costs and effectiveness were discounted at a rate of 3%, in accordance with the recommended guidelines by the WHO.^17^ Currently, China lacks a specific policy regarding the establishment of cost-effectiveness thresholds for vaccines. Consequently, various multiples of gross domestic product (GDP) per capita (1 GDP = $12,680.83) were employed as thresholds to evaluate the cost-effectiveness of PPSV23.^18^ The model was programmed in Microsoft Excel 365 (Microsoft Corporation; Redmond, WA, USA).

### 2.2 Model inputs

The key model parameters were outlined in Table 1. Epidemiological data, encompassing age-dependent incidence rates and mortality rates, and the sequelae of survivors, are derived from the most up-to-date Global Burden of Disease 2021 data or studies conducted on Asian populations.^2^^,^^19^, ^20^, ^21^ Approximately 29% of patients with CAP necessitated hospitalization and the PPSV23 vaccine provided coverage against a total of 79.49% of pathogenic serotypes associated with pneumococcal infections in Chinese studies.^9^^,^^22^ According to the existing literature^23^^,^^24^, the efficacy data regarding age- and time-dependent suggest a gradual decline in the effectiveness of PPSV23 against CAP and IPD with increasing age and duration since vaccination. Research findings indicate that the vaccine’s protective power diminishes to zero after 15 years of vaccination. In parallel, the Advisory Committee on Immunization Practices recommends that PPSV23 revaccination may be considered in selected high-risk populations, such as individuals with immunocompromising conditions, while routine revaccination of immunocompetent adults is not universally recommended.^25^ In light of the documented waning of vaccine effectiveness and the absence of unified adult pneumococcal vaccination guidelines in China, the base-case analysis of our model incorporates a hypothetical revaccination scenario assuming a second dose of PPSV23 administered 15 years after the initial vaccination to explore long-term protection over the adult lifespan. To assess the robustness and generalizability of this assumption, a scenario analysis assuming a single-dose vaccination strategy without revaccination was also conducted. The disease risk in vaccinated individuals was determined for each health state using the Equation (1)^26^:

(1) $$\begin{array}{ccc} & & \mathrm{Dise}\mathrm{ase}\;\mathrm{risk}\;\mathrm{in}\;\mathrm{vacc}\mathrm{inat}\mathrm{ed}\;\mathrm{indi}\mathrm{vidu}\mathrm{als}\\ & & \;=\mathrm{prob}\mathrm{abil}\mathrm{ity}\;\mathrm{among}\;\mathrm{unva}\mathrm{ccin}\mathrm{ated}\;\mathrm{indi}\mathrm{vidu}\mathrm{als}\\ & & \;\times \;\left[1-\left(\mathrm{vacc}\mathrm{ine}\;\mathrm{effe}\mathrm{ctiv}\mathrm{eness}\times \mathrm{sero}\mathrm{type}\;\mathrm{cove}\mathrm{rage}\right)\right] \end{array}$$

**Table 1:** Input data for the cost-effectiveness model.

Parameter	Base case (Range)	Distribution	Source
Clinical data			
Incidence (cases/100,000 persons)			
Incidence of CAP			^19^
50–54 years	183.09 (146.47–219.70)	Beta	
55–59 years	221.83 (177.46–266.19)	Beta	
60–64 years	369.05 (295.24–442.86)	Beta	
65–69 years	604.89 (483.91–725.87)	Beta	
70–74 years	821.06 (656.85–985.28)	Beta	
75–79 years	1054.26 (843.41–1265.11)	Beta	
80–84 years	2302.72 (1842.17–2763.26)	Beta	
≥ 85 years	5298.42 (4238.73–6358.10)	Beta	
Incidence of meningitis			^19^
50–54 years	0.15 (0.12–0.18)	Beta	
55–59 years	0.18 (0.15–0.22)	Beta	
60–64 years	0.25 (0.20–0.30)	Beta	
65–69 years	0.34 (0.27–0.41)	Beta	
70–74 years	0.52 (0.41–0.62)	Beta	
75–79 years	0.78 (0.62–0.94)	Beta	
80–84 years	1.38 (1.10–1.65)	Beta	
≥ 85 years	2.87 (2.30–3.45)	Beta	
Incidence of bacteremia			^2^
50–54 years	0.90 (0.72–1.08)	Beta	
55–59 years	0.90 (0.72–1.08)	Beta	
60–64 years	0.90 (0.72–1.08)	Beta	
65–69 years	2.50 (2.00–3.00)	Beta	
70–74 years	2.50 (2.00–3.00)	Beta	
75–79 years	6.10 (4.88–7.32)	Beta	
80–84 years	6.10 (4.88–7.32)	Beta	
≥ 85 years	12.30 (9.84–14.76)	Beta	
Proportion of hospitalized CAP (%)	29.29 (23.43–35.15)	Beta	^9^
Mortality (%)			
All-cause mortality rate			^21^
50–54 years	0.30	—	
55–59 years	0.45	—	
60–64 years	0.74	—	
65–69 years	1.18	—	
70–74 years	2.02	—	
75–79 years	3.57	—	
80–84 years	6.24	—	
85–90 years	10.32	—	
90–94 years	16.26	—	
≥ 95 years	20.70	—	
Mortality of hospitalized CAP			^2^
50–54 years	7.6 (6.08–9.12)	Beta	
55–59 years	7.6 (6.08–9.12)	Beta	
60–64 years	7.6 (6.08–9.12)	Beta	
65–69 years	7.8 (6.24–9.36)	Beta	
70–74 years	7.8 (6.24–9.36)	Beta	
75–79 years	10.9 (8.72–13.08)	Beta	
80–84 years	10.9 (8.72–13.08)	Beta	
≥ 85 years	13.6 (10.88–16.32)	Beta	
Mortality of meningitis			^2^
50–54 years	5.3 (4.24–6.36)	Beta	
55–59 years	5.3 (4.24–6.36)	Beta	
60–64 years	5.3 (4.24–6.36)	Beta	
65–69 years	5.3 (4.24–6.36)	Beta	
70–74 years	5.3 (4.24–6.36)	Beta	
75–79 years	12.5 (10–15)	Beta	
80–84 years	12.5 (10–15)	Beta	
≥ 85 years	12.5 (10–15)	Beta	
Mortality of bacteremia			^2^
50–54 years	7.8 (6.24–9.36)	Beta	
55–59 years	7.8 (6.24–9.36)	Beta	
60–64 years	7.8 (6.24–9.36)	Beta	
65–69 years	8.2 (6.56–9.84)	Beta	
70–74 years	8.2 (6.56–9.84)	Beta	
75–79 years	16.1 (12.88–19.32)	Beta	
80–84 years	16.1 (12.88–19.32)	Beta	
≥ 85 years	18.7 (14.96–22.44)	Beta	
Mortality from sequelae (%)	5 (4–6)	Beta	^2^
Sequelae of survivors (%)			^20^
Bacteremia result in sequelae	7 (5.6–8.4)	Beta	
Meningitis result in sequelae	30 (24–36)	Beta	
CAP result in sequelae	2.7 (2.16–3.24)	Beta	
Vaccine serotype coverage (%)			^22^
Coverage of PPSV23	79.49 (63.59–95.39)	Beta	
Vaccine efficacy			
Vaccine efficacy of PPSV23-CAP (50 years)			^23^
1 year	0.23 (0.2–0.24)	Beta	
3 years	0.22 (0.19–0.24)	Beta	
5 years	0.21 (0.17–0.23)	Beta	
7 years	0.15 (0.1–0.19)	Beta	
10 years	0.05 (0–0.08)	Beta	
15 years	0 (0–0.05)	—	
Vaccine efficacy of PPSV23-CAP (65 years)			^23^
1 year	0.2 (0.15–0.23)	Beta	
3 years	0.18 (0.13–0.21)	Beta	
5 years	0.15 (0.08–0.2)	Beta	
7 years	0.08 (0.03–0.12)	Beta	
10 years	0 (0–0.02)	—	
15 years	0 (0–0.02)	—	
Vaccine efficacy of PPSV23-IPD (50 years)			^24^
1 year	0.93 (0.8–0.95)	Beta	
3 years	0.89 (0.74–0.95)	Beta	
5 years	0.85 (0.66–0.9)	Beta	
7 years	0.6 (0.4–0.75)	Beta	
10 years	0.2 (0–0.3)	Beta	
15 years	0 (0–0.2)	—	
Vaccine efficacy of PPSV23-IPD (65 years)			^24^
1 year	0.8 (0.6–0.9)	Beta	
3 years	0.73 (0.5–0.83)	Beta	
5 years	0.58 (0.31–0.8)	Beta	
7 years	0.33 (0.13–0.48)	Beta	
10 years	0 (0–0.1)	—	
15 years	0 (0–0.1)	—	
Vaccination cost data ($)			^26^
Cost of PPSV23 per dose	28.81 (23.05–34.57)	Gamma	
Disease burden cost data ($)			
Cost of outpatient pneumonia	129 (103.2–154.8)	Gamma	^27^
Cost of inpatient pneumonia	2735 (2188–3282)	Gamma	^27^
Cost of meningitis	7105.67 (5684.54–8526.81)	Gamma	^28^
Cost of bacteremia	7596.74 (6077.39–9116.09)	Gamma	^28^
Cost of sequelae per year	1889.7 (1511.76–2267.64)	Gamma	^9^
Daily wage for caregiver	34.77 (27.81–41.72)	Gamma	^18^
Duration of illness (days)			^29^
Days lost of IPD hospitalization for bacteremia	22 (17.6–26.4)	Gamma	
Days lost of IPD hospitalization for meningitis	32 (25.6–38.4)	Gamma	
Days lost of CAP hospitalization	16 (12.8–19.2)	Gamma	
Days lost of CAP outpatient care	7 (5.6–8.4)	Gamma	
Utility scores			
Utility of normal population			^30^
50–54 years	0.78 (0.62–0.94)	Beta	
55–59 years	0.76 (0.61–0.91)	Beta	
60–64 years	0.74 (0.59–0.89)	Beta	
65–69 years	0.71 (0.57–0.85)	Beta	
70–74 years	0.69 (0.55–0.83)	Beta	
75–79 years	0.68 (0.54–0.82)	Beta	
80–84 years	0.66 (0.53–0.79)	Beta	
≥ 85 years	0.65 (0.52–0.78)	Beta	
Utility of outpatient pneumonia	0.61 (0.49–0.73)	Beta	^31^
Utility of inpatient pneumonia	0.56 (0.45–0.67)	Beta	^31^
Utility of IPD	0.2 (0.16–0.24)	Beta	^32^
Utility of disabled	0.4 (0.32–0.48)	Beta	^33^
Discount rate (%)	3 (0–8)	Beta	^17^

*Abbreviations*: PPSV23, 23-valent pneumococcal polysaccharide vaccine; CAP, community-acquired pneumonia; IPD, invasive pneumococcal disease.

The direct medical costs encompass expenses for vaccination, outpatient care, hospitalization, and pneumococcal illness. The price of PPSV23 per dose was $28.81, derived from the average bid-winning price in China.^27^ Based on the literature published by Chinese scholars^9^^,^^28^^,^^29^, we conducted a cost assessment of PD. Indirect costs encompass productivity losses attributed to vaccination, PD, premature mortality, and caregivers. The duration of illness was extracted from the published literature^30^, while the average daily wage was obtained from the National Bureau of Statistics of China.^18^ The productivity loss caused by premature death is calculated until the retirement age of 60 years. The age-specific EuroQol-5 dimension values for establishing baseline utilities in a Chinese population were obtained from Sun et al.^31^ The utility of 0.61, 0.56, 0.20 and 0.40 were applied for outpatient pneumonia, inpatient pneumonia, IPD and sequelae, respectively^32^, ^33^, ^34^ The utility scores for CAP and IPD were applied throughout the duration of illness and subsequently converted to baseline population utilities, while the utility scores for long-term sequelae were applied for the remaining lifespan of the patient.

### 2.3 Base-case analysis

The model output encompasses the cumulative incidence rate, cost, life-year (LY) and quality-adjusted life year (QALY) in terms of both disease burden and economic aspects. The incremental cost-effectiveness ratio (ICER) was calculated as the additional costs per gained QALY between the PPSV23 vaccination and no vaccination, and both LY and QALY as effectiveness were calculated. We set 1 GDP as the threshold for willingness-to-pay (WTP) threshold in the base-case analysis, and when ICER is less than WTP, the PPSV23 was considered cost-effective in accordance with the recommendations outlined in the China guidelines for pharmacoeconomic evaluations 2020.^35^ The incremental net health benefits (INHB) and incremental net monetary benefits (INMB) were also incorporated into our analyses using the Equations (2) and (3)^36^:

(2) $$INHB\left(\lambda \right)=\left(\mu E_{1}-\mu E_{0}\right)-\left(\mu C_{1}-\mu C_{0}\right)/\lambda =\Delta E-\Delta C/\lambda$$

(3) $$\begin{array}{c} \mathrm{INMB}\left(\lambda \right)=\left(\mu E_{1}-\mu E_{0}\right)\times \lambda -\left(\mu C_{1}-\mu C_{0}\right)=\Delta E\times \lambda -\Delta C \end{array}$$

Where μ*C*_i_ and μ*E*_i_ represented the costs and QALYs of PPSV23 for i = 1 or no vaccination for i = 0, respectively, while *λ* denoted the WTP threshold.

### 2.4 Sensitivity and scenario analyses

We performed deterministic and probabilistic sensitivity analyses (DSA and PSA) to evaluate the robustness and cost-effectiveness of PPSV23, by varying all probabilities, vaccine efficacy, costs, utilities, discount rate, and duration of illness. The parameters in DSA were adjusted both upwards and downwards by 20% of their original values, with the exception of those for which recommended values were available. To assess the combined impact of a set of parameters (e.g., incidence of CAP), we packaged the age- and time-dependent parameters separately and applied the upper or lower bounds to the parameters within each group simultaneously in the DSA. DSA involved sequential parameter modifications, whereas PSA entailed concurrent parameter resampling from predetermined distributions of input values. We employed the beta distribution to model probabilities, ratios, utilities, discount rate, and vaccine efficacy, while the gamma distribution was utilized for costs and duration of illness. The standard error is calculated as the difference between the upper and lower limits, divided by 3.92. After conducting 1000 iterations of Monte Carlo simulation, repeated sampling was performed to obtain the PSA results. Subsequently, we use the expected value of perfect information (EVPI) to quantify the losses caused by suboptimal decisions based on incomplete information. The EVPI was computed using the Equation (4)^37^:

(4) $$\begin{array}{ccc} \mathrm{EVPI} & = & \text{Average}\;\left[\max\left(\mathrm{NMB},\;\mathrm{PPSV}23,\;\text{no}\;\;\text{vaccination}\;\right)\right]\\ & & -\;\max\left[\;\text{Average}\;\left(\mathrm{NMB},\;\mathrm{PPSV}23,\;\text{no}\;\;\text{vaccination}\right)\right] \end{array}$$

We estimated the probability of PPSV23 to be cost-effective under different WTP thresholds in various provinces and cities in China^18^, while conducting a price simulation by varying the cost per dose of PPSV23 from $0 to $70. Moreover, given that the package insert does not explicitly require revaccination with PPSV23 for all populations, we also simulated a scenario where only one dose of PPSV23 is administered at age 50.

### 2.5 Safety assessment

The Vaccine Adverse Event Reporting System (VAERS) is a nationwide surveillance program jointly administered by the Centers for Disease Control and Prevention and the Food and Drug Administration.^38^ It serves as a repository for spontaneous reports of AEs occurring after vaccination. We conducted a comprehensive analysis of USA reports submitted to the VAERS between 2014 and 2024, aiming to provide an in-depth characterization of the safety profile associated with PPSV23 vaccination. Reports are classified as serious according to the Code of Federal Regulations if they result in any of the following outcomes: death, permanent disability, life threatening, hospitalized, existing hospitalization prolonged, congenital anomaly or birth defect.^39^ The coding of symptoms in VAERS reports were standardized using MedDRA 27.0 preferred term (PT) to ensure consistency and facilitate mapping to the system organ class level, and a single VAERS report may be associated with multiple PTs. We compiled a comprehensive list of the most frequently reported MedDRA PTs for both serious and non-serious reports. We employed the empirical Bayesian geometric mean (EBGM) data mining approach to identify MedDRA PTs that exhibited a disproportional reporting pattern (i.e., higher frequency of reports compared to other vaccines in VAERS) following the administration of PPSV23.^40^ Disproportionate reporting of AEs cannot establish causality between vaccination and AEs, but it can serve as a valuable tool for identifying potential vaccine safety concerns or signals that require further investigation. The VAERS data management and analysis were performed using MySQL 8.0.

## 3 Results

### 3.1 Base-case analysis

The results of the base-case analysis were shown in Table 2. Compared to no vaccination, PPSV23 vaccination is projected to reduce the disease burden, with cumulative probabilities of CAP, hospitalization, IPD, sequelae and death following pneumococcal disease decreasing by 1.09%, 0.32%, 0.02%, 0.01% and 0.03%, respectively. Although the medical costs associated with disease were lower for PPSV23, the overall expenses were higher due to the vaccination costs of PPSV23 compared to no vaccination. Additionally, an additional expenditure of approximately $27.97 per person was incurred for PPSV23. Compared with no vaccination, the average QALY and LY gain per person were 0.00216 and 0.00291, respectively. At the current PPSV23 vaccine price, the estimated ICERs for PPSV23 compared with no vaccination are $12,937.07 per QALY gained and $9,622.25 per LY gained, respectively. The INHB was estimated to be −0.00004 QALYs, while the INMB amounted to −$0.55, based on a WTP threshold of $12,680.83 per QALY. Assuming a study population of 100,000 individuals, the administration of PPSV23 resulted in the prevention of 1,115 cases and 35 deaths due to pneumococcal infection compared to no vaccination. This intervention incurred an additional cost of $2,796,745 but led to an increase of 216 QALYs and 291 LYs.

**Table 2:** Results of cost-effectiveness analysis of PPSV23 compared to no vaccination.

Strategy	No vaccination	PPSV23
Case		
Probability of CAP (%)	37.77	36.69
Outpatient pneumonia	26.71	25.94
Inpatient pneumonia	11.06	10.75
Probability of IPD (%)	0.138	0.118
Meningitis	0.023	0.020
Bacteremia	0.115	0.098
Probability of sequelae (%)		
Inpatient pneumonia	0.299	0.290
Meningitis	0.007	0.006
Bacteremia	0.008	0.007
Probability of Death (%)		
CAP	1.251	1.227
IPD		
Meningitis	0.00238	0.00222
Bacteremia	0.01677	0.01542
Sequelae	0.18226	0.17338
Cost-effectiveness		
Cost ($)	264.02	291.99
QALYs (QALY)	14.86685	14.86902
LYs (year)	20.33848	20.34139
Incremental cost	—	27.97
Incremental QALY	—	0.00216
Incremental LY	—	0.00291
Incremental cost per QALY	—	12,937.07
Incremental cost per LY	—	9622.25
INHB (QALY)	—	−0.00004
INMB ($)	—	−0.55
EVPI/person ($)	—	4.16

*Abbreviations*: PPSV23, 23-valent pneumococcal polysaccharide vaccine; CAP, community-acquired pneumonia; IPD, invasive pneumococcal disease; INHB, incremental net health benefit; INMB, incremental net monetary benefit; QALY, quality-adjusted life-years; LY, life-year; EVPI, expected value of perfect information.

### 3.2 Sensitivity and scenario analyses

**Fig. 1:**
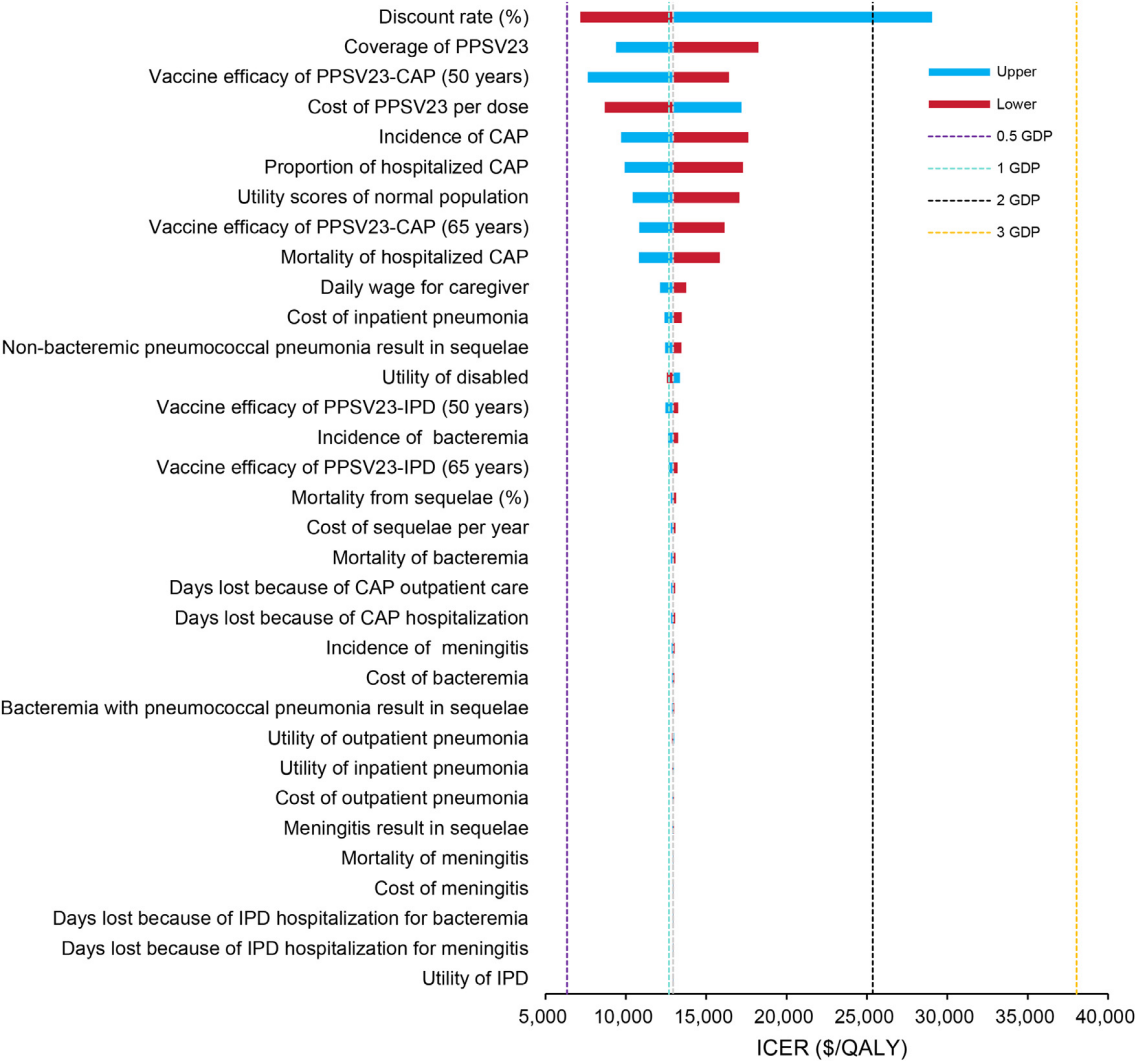
The one-way sensitivity analysis comparing the impact of PPSV23 vaccination to no vaccination.

*Abbreviations*: GDP, gross domestic product; PPSV23, 23-valent pneumococcal polysaccharide vaccine; CAP, community-acquired pneumonia; IPD, invasive pneumococcal disease; ICER, incremental cost-effectiveness ratio; QALY, quality-adjusted life year.

**Fig. 2:**
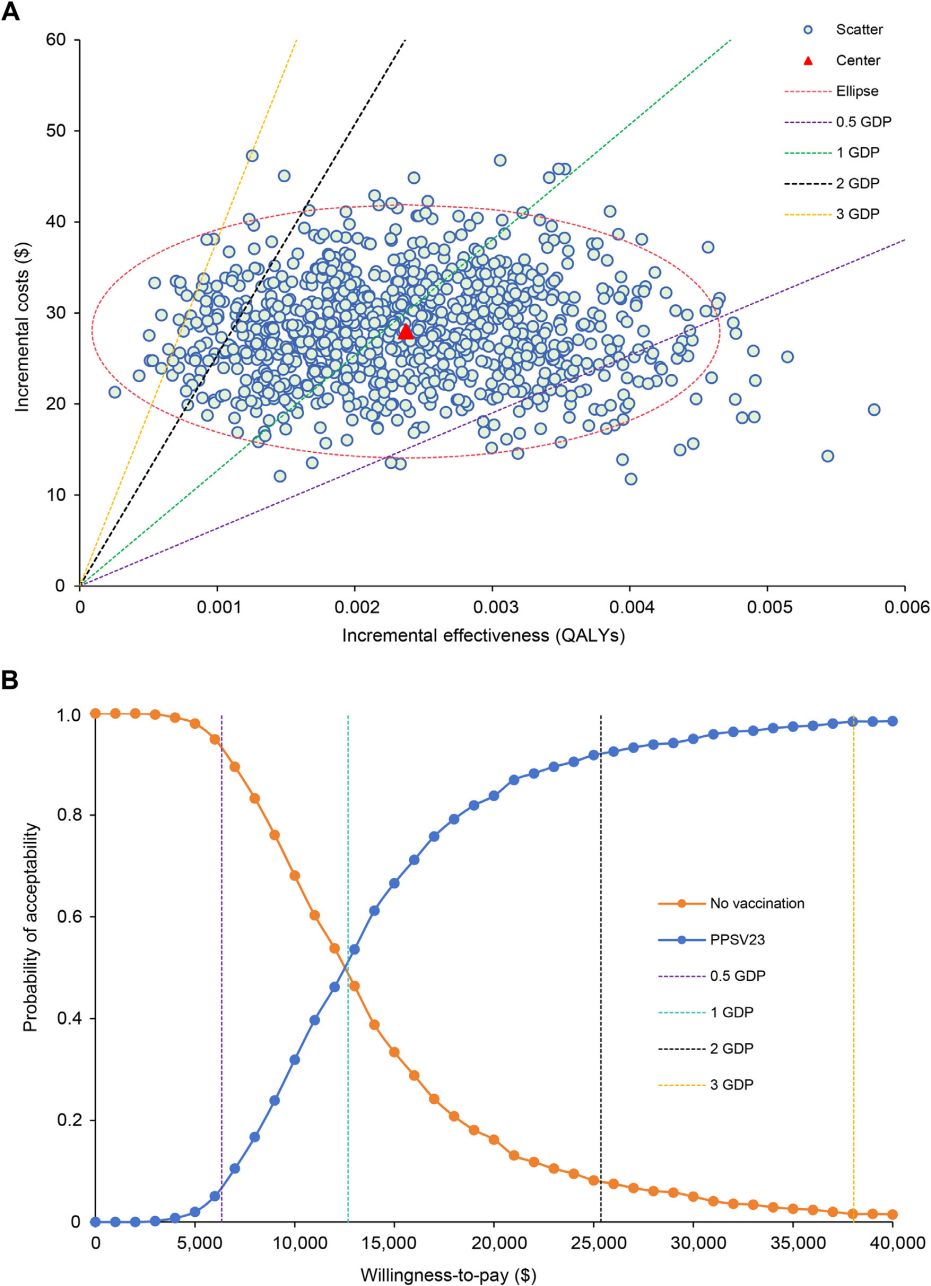
Probabilistic sensitivity analyses of PPSV23 vs. no vaccination. (A) Scatter diagram. (B) Acceptability curves.

*Abbreviations*: GDP, gross domestic product; QALY, quality-adjusted life year; PPSV23, 23-valent pneumococcal polysaccharide vaccine.

We presented a tornado diagram illustrating the sensitivity analysis results of DSA (Fig. 1). The ICER demonstrated the highest level of sensitivity to variations in the discount rate, serotype coverage of PPSV23, vaccine efficacy of PPSV23-CAP (50 years), cost per dose of PPSV23, and incidence rate of CAP. The fluctuation of these parameters, however, failed to yield an ICER below the threshold of 0.5 GDP or above the threshold of 3 GDP (excluding discount rate). With all iterations falling in the northeast quadrant (Fig. 2A), PPSV23 exhibited probabilities of being cost-effective compared to no vaccination of 6.90%, 51.70%, 92.10%, and 98.40% at ceiling thresholds of 0.5 GDP, 1 GDP, 2 GDP, and 3 GDP, respectively. Based on the PSA results, we plotted a cost-effectiveness acceptability curve (Fig. 2B) and the expected value of uncertainty measured by EVPI was $4.16 per person. Furthermore, we conducted an analysis to assess the probability of being cost-effective and corresponding EVPI for PPSV23 across 32 provinces and municipalities in China, with detailed results provided in Supplementary Table S1 and Supplementary Fig. S2.

**Fig. 3:**
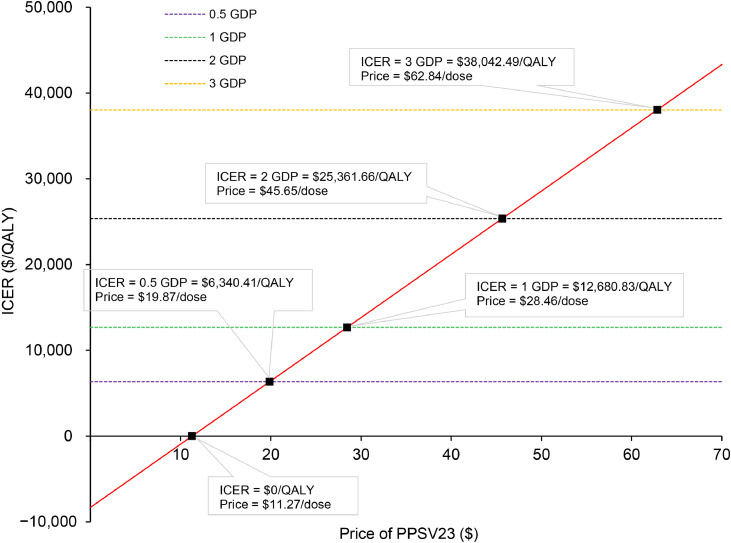
Results of price simulation.

*Abbreviations*: GDP, gross domestic product; ICER, incremental cost-effectiveness ratio; QALY, quality-adjusted life year; PPSV23, 23-valent pneumococcal polysaccharide vaccine.

The results of price simulation were shown in Fig. 3. PPSV23 was favored when the price was less than $19.87, $28.46, $45.65, $62.84 per dose at the WTP thresholds of 0.5 GDP, 1 GDP, 2 GDP, and 3 GDP, respectively. When the price of PPSV23 drops to $11.27 per dose, the ICER reaches 0, indicating that the economic benefits derived from vaccination are equal to the costs incurred. The ICERs for PPSV23 with prices ranging from $0 to $70 per dose are summarized in Supplementary Table S2, and scatter plots for the PSA can be found in the Supplementary Fig. S3. The cost-effectiveness analysis presented in Supplementary Table S3 examines the impact of administering a single dose of PPV23 to individuals aged 50 years. In comparison to the base-case findings, vaccinating with only one dose results in reduced costs but also leads to fewer reductions in pneumococcal cases and death, yielding a higher ICER of $13,471.12 per QALY.

### 3.3 Safety of PPSV23

VAERS received a total of 1,434,320 AE reports in the US from January 01, 2014, through April 26, 2024, including 29,536 reports involving the receipt of PPSV23 vaccination. The detailed information categorized by distinct age cohorts was presented in Supplementary Table S4, while the number of reports for each state was shown in Supplementary Fig. S4. The states with the most VAERS reports were California (1,871), Pennsylvania (1,822), Florida (1,523), Texas (1,454), and New York (1,116). Female accounted for 17,018 (57.62%) reports, while male accounted for 6,995 (23.68%) reports. In reports where age and time-to-onset were recorded, the median age was 66 years (interquartile range, IQR: 55–71 years), with a median time-to-onset of 0 days (IQR: 0–1 days). Among them, the proportion of reports from individuals aged ≥ 50 years is as high as 80.81%. PPSV23 was given alone in 97.53% of the reports, while an overall majority of 95.29% of the reports indicated non-serious outcomes. Despite the identification of 61 reports documenting deaths subsequent to PPSV23 vaccination, neither the available information within these reports nor their accompanying documentation provided any indication that PPSV23 was causally related or contributed to these fatalities. The Table 3 presents the 10 most frequently reported MedDRA PTs for both serious and non-serious reports. Among non-serious reports, injection site erythema, erythema, and injection site swelling were the most commonly documented symptoms. Pyrexia, pain in extremity, and injection site pain were frequently reported symptoms in serious reports. Data mining results revealed a significant increase in disproportional reporting of 95 PTs (EBGM05 > 2) across 7 primary system organ classes following PPSV23 vaccination, as shown in Supplementary Table S5. These signals are almost identical to the AEs listed in the PPSV23 package insert.

**Table 3:** Most commonly reported PT after vaccination of PPSV23 in reports submitted to VAERS, USA, January 01, 2014–April 26, 2024.

All reports, PT	*n* (%)	Non-serious reports, PT	*n* (%)	Serious reports, PT	*n* (%)
Injection site erythema	5892 (19.95)	Injection site erythema	5675 (20.16)	Pyrexia	411 (29.53)
Erythema	5389 (18.25)	Erythema	5192 (18.45)	Pain in extremity	247 (17.74)
Injection site swelling	5178 (17.53)	Injection site swelling	4977 (17.68)	Injection site pain	242 (17.39)
Injection site pain	5014 (16.98)	Injection site pain	4772 (16.96)	Injection site erythema	217 (15.59)
Pain in extremity	4645 (15.73)	Pain in extremity	4398 (15.63)	Pain	215 (15.45)
Pyrexia	4233 (14.33)	Peripheral swelling	3836 (13.63)	Injection site swelling	201 (14.44)
Peripheral swelling	4009 (13.57)	Pyrexia	3822 (13.58)	Cellulitis	200 (14.37)
Pain	3689 (12.49)	Pain	3474 (12.34)	Erythema	197 (14.15)
Injection site warmth	2478 (8.39)	Injection site warmth	2417 (8.59)	White blood cell count increased	176 (12.64)
Skin warm	2295 (7.77)	Skin warm	2237 (7.95)	Peripheral swelling	173 (12.43)

*Abbreviations*: PT, preferred term; PPSV23, 23-valent pneumococcal polysaccharide vaccine; VAERS, vaccine adverse event reporting system.

## 4 Discussion

PPSV23 has been utilized in China for many years, yet it remains excluded from the national immunization program. The burden of disease caused by PD is significant.^41^ While incorporating PD into the national immunization program is the optimal strategy^42^, we aim to promote higher PPSV23 vaccination coverage by evaluating its cost-effectiveness and safety to enhance public awareness and trust in the vaccine. Although the effectiveness of PPSV23 in preventing PD among the elderly has been widely confirmed, the vaccination coverage among the elderly in China is less than 3.7%^43^, with the rate even lower among those aged 50 years.^44^ This is significantly lower than in western countries, such as the USA, where vaccination rates among the elderly are as high as 60%.^45^ While several studies have evaluated the cost-effectiveness of PPSV23 vaccination among individuals aged 60 and older in China, this study represents the first cost-effectiveness analysis targeting the 50-year-old population. Furthermore, we examined data from the USA VAERS over the past decade to assess the real-world safety profile of PPSV23. Given that vaccine efficacy diminishes over time, in accordance with the guidelines provided in the manual, our research design involves administering a booster dose at age 50 and another at age 65. Moreover, we have also evaluated the scenario of administering only a single dose at age 50.

The study found that both one and two doses of PPSV23 are cost-effective options compared to no vaccination for adults aged 50 years in China, with a WTP threshold of 3 GDP, approximately $38,042.49 per QALY, as recommended by China Guidelines for Pharmacoeconomic Evaluations 2020.^35^ When the price of PPSV23 drops to $11.27 per dose, it becomes the dominant strategy, as the cost of PD avoided by vaccination offsets the cost of the vaccination. The one-way sensitivity analysis found that the serotype coverage and vaccine efficacy of PPSV23 were significant factors influencing the model’s economic outcomes, although these factors did not reverse the model’s economic results, which is consistent with other studies.^9^^,^^11^ However, the most significant factor affecting the model’s results was the discount rate. This may be due to the fact that as the discount rate increases, the present value of future health benefits and savings in medical costs decreases, especially in vaccine programs with significant long-term immunity effects, where the impact of the discount rate on the results is particularly pronounced.^46^ Compared to previous PPSV23 cost-effectiveness analyses in China for individuals over 60^9^^,^^11^, our study not only accounts for differences in the incidence of pneumococcal disease and vaccine efficacy between the different populations, but also considers the prevention of pneumococcal disease in adults, which avoids both acute and long-term productivity losses due to disability or premature mortality. Based on the fact that the effectiveness of the PPSV23 vaccine decreases to zero 15 years after administration, we recommend revaccination to avoid neglecting its residual efficacy. Additionally, some studies suggest that the cost-effectiveness of revaccination every 10–15 years is significantly better than that of every 5 years^9^^,^^47^, and vaccination with PPSV23 at age 50 results in a lower ICER and prevents more PD cases. The small differences in study results can be attributed to various factors, but most studies from different countries and regions indicate that the PPSV23 vaccine is cost-effective. With the future introduction of other pneumonia vaccines in the domestic market, such as the 15-valent and 20-valent PCVs, more economic evaluations of vaccine combinations will become necessary. Furthermore, the experience from bulk procurement of pharmaceuticals can be leveraged to reduce vaccine prices.^48^ The generalizability of our findings should be interpreted in the context of adult pneumococcal vaccination policies, which vary across countries and risk groups. In this study, revaccination at age 65 was modelled as a hypothetical policy scenario to explore long-term protection under waning vaccine effectiveness, rather than to reflect current routine practice. Importantly, scenario analyses assuming a single-dose PPSV23 strategy without revaccination yielded consistent directional results, suggesting that the overall conclusions were not driven by the revaccination assumption and may be interpreted according to local vaccination policies.

In addition to the cost-effectiveness of vaccines, public concern about vaccine safety also significantly impacts adult vaccination rates.^43^^,^^49^ Our study found that the majority of AE reports following PPSV23 vaccination are mild, with most being related to the injection site, and no new potential AEs were identified. These reactions typically resolve within a short period, usually within one day. Death reports associated with PPSV23 vaccination are extremely rare, and available VAERS reports do not provide evidence suggesting a causal association between PPSV23 vaccination and death. Given that PPSV23 is primarily administered to older adults, deaths temporally reported after vaccination are expected to occur coincidentally due to underlying comorbidities and background mortality in these populations. In a latest self-controlled risk interval study, Yoon et al.^15^ found there was no appreciable increase in risk for most cardiovascular, neurological, or immunological AEs following PPSV23 vaccination in older adults. Numerous studies and extensive clinical experience have consistently demonstrated the safety of PPSV23 vaccination, and our VAERS data mining study further strengthens this evidence. It is worth noting that our safety assessment used VAERS, a USA-based passive surveillance system. Differences in population characteristics, background event rates, healthcare utilization, and reporting behaviors between the USA and China may limit the direct applicability of these findings to the Chinese setting. Moreover, VAERS data are subject to underreporting, stimulated reporting, and incomplete clinical verification, which means that disproportionality analyses (e.g., EBGM) cannot establish causality or provide incidence estimates. As such, our VAERS analysis should be interpreted as signal detection and general safety reassurance rather than China-specific risk quantification. Future research should incorporate Chinese post-marketing surveillance data, such as those from the Chinese Center for Disease Control and Prevention (https://www.chinacdc.cn/en/), to generate more locally relevant safety evidence. Given the inherent limitations of passive surveillance systems, VAERS data were not used to quantify AE risks in the economic model.

The present study is subject to several limitations. First, potential uncertainties may exist in the data sources and assumptions utilized. For instance, we made the assumption that individuals who experienced permanent disability would remain in a disabled health status until death. However, when considering varying input parameters within reasonable ranges, the impact on results was relatively minimal. Overall, the probability of sequelae is relatively low and has negligible effects on outcomes and study conclusions. Due to the lack of long-term effectiveness data on PPSV23 vaccine in China, we incorporated effectiveness data from other countries into our model. However, considering the robustness of our analysis results, we anticipate that these effectiveness data will have a predictably small impact on the outcomes. Second, our model did not take into account herd immunity and adverse reactions to vaccination, resulting in underestimation and overestimation of the vaccine’s effectiveness. However, the effectiveness of herd immunity is relatively weaker among older adults. Additionally, our analysis of VAERS data confirmed that most adverse reactions caused by PPSV23 are mild. Although these events are generally self-limited and resolve within a few days, their exclusion may lead to a slight underestimation of total vaccination-related costs. Nevertheless, given their low severity, short duration, and limited economic impact, this simplification is unlikely to materially influence the overall cost-effectiveness results. Third, due to the inherent limitations of VAERS, results derived from data mining analyses require cautious interpretation and should be considered alongside evidence from pre-licensure clinical trials and other post-licensure studies. Finally, our model is based on cohorts aged 50 years old and tracked throughout their lifetimes, without considering cohorts of varying ages. Therefore, caution must be exercised when generalizing the results to other age groups. Nevertheless, we contend that the model and methodology employed in this study can serve as a valuable reference.

## 5 Conclusion

Our analysis reveals that among individuals aged 50 in China, the PPSV23 vaccine not only effectively prevents more pneumococcal-related diseases compared to no vaccination but also exhibits substantial cost-effectiveness at higher WTP thresholds (e.g., 2–3 × GDP per capita). Although these conclusions are sensitive to assumptions regarding discount rate, serotype coverage of PPSV23, vaccine efficacy of PPSV23 and cost per dose of PPSV23, the overall economic conclusions remain robust across scenario and sensitivity analyses. The safety data for PPSV23 derived from VAERS align with the results from pre-licensure clinical trials and other post-licensure studies. These findings provide valuable insights for optimizing and implementing vaccination strategies.

## CRediT authorship contribution statement

**Yamin Shu:** Writing – review & editing, Writing – original draft, Software, Methodology, Formal analysis, Data curation, Conceptualization. **Shuhua Tan:** Writing – original draft, Methodology, Formal analysis, Software, Writing – review & editing. **Yao Chen:** Writing – original draft, Data curation. **Yiling Ding:** Writing – original draft, Software, Formal analysis, Writing – review & editing. **Pingping Xu:** Writing – review & editing, Writing – original draft, Conceptualization. **Qilin Zhang:** Writing – review & editing, Writing – original draft, Supervision, Funding acquisition, Conceptualization.

## Informed consent

Not applicable.

## Organ donation

Not applicable.

## Ethical statement

This study used only publicly available, de-identified data and did not involve human subjects; therefore, ethical approval was not required.

## Data availability statement

All data generated or analysed during this study are included in this published article and its supplementary information files. The data that support the findings of this study are available on request from the corresponding author.

## Animal treatment

Not applicable.

## Generative AI

Not applicable.

## Funding

This study was supported by grants from Project of the Health Commission of Hubei Province (No. WJ2025M193), The Talent Project established by Chinese Pharmaceutical Association Hospital Pharmacy Department (No. CPA-Z05-ZC-2023-003), and Dawning Program of Wuhan Knowledge Innovation Special Project (No. 2023020201020501).

## Declaration of competing interest

The authors declare that they have no competing interests.

## Appendix Supplementary materials

Image, application 1
