# Supplementary material for: Cost-effectiveness analysis of 23-valent pneumococcal polysaccharide vaccine for adults in China
Source: Infect Med (Beijing). 2026 Apr 27;5(2):100258. doi: 10.1016/j.imj.2026.100258 (PMC13196514; doi:10.1016/j.imj.2026.100258)

**Supplementary Materials**

**Supplementary Table S1. The probability of cost-effectiveness and EVPI in different provinces and cities of China.**

**Supplementary Table S2. Summary of the ICERs for PPSV23 at different prices.**

**Supplementary Table S3. Only one dose of PPSV23 at age 50.**

**Supplementary Table S4. Characteristics of PPSV23 reports submitted to VAERS, United States, January 01 2014-April 26 2024.**

**Supplementary Table S5. MedDRA PTs with a disproportionality score EBGM05>2.0 grouped by SOC for PPSV23 in VAERS, United States, January 01 2014-April 26 2024.**

**Supplementary Figure S1. Model Structure.**

**Supplementary Figure S2. The probability of cost-effectiveness for PPSV23 by province.**

**Supplementary Figure S3. Scatter plots for PSA with different prices.**

**Supplementary Figure S4. VAERS reports from various states.**

**Supplementary Table S1.** **The probability of cost-effectiveness and EVPI in different provincial-level administrative units of China.**

| Provincial-level administrative units | WTP ($) | Probability of being cost-effective (%) | EVPI ($/person) |
| --- | --- | --- | --- |
| Anhui | 10,903 | 39.40 | 3.74 |
| Beijing | 28,421 | 93.90 | 0.34 |
| Chongqing | 13,359 | 56.70 | 3.65 |
| Fujian | 18,429 | 80.00 | 1.48 |
| Gansu | 6793 | 9.30 | 0.45 |
| Guangdong | 15,182 | 67.80 | 2.59 |
| Guangxi | 7664 | 14.30 | 0.84 |
| Guizhou | 7688 | 14.40 | 0.85 |
| Hainan | 10,353 | 34.50 | 3.08 |
| Hebei | 8420 | 20.70 | 1.31 |
| Heilongjiang | 7317 | 12.00 | 0.66 |
| Henan | 8525 | 21.40 | 1.39 |
| Hubei | 13,558 | 57.60 | 3.51 |
| Hunan | 10,776 | 38.30 | 3.59 |
| Jiangsu | 21,356 | 87.30 | 0.94 |
| Jiangxi | 10,106 | 32.90 | 2.81 |
| Jilin | 8194 | 18.40 | 1.15 |
| Liaoning | 10,233 | 33.90 | 2.95 |
| Inner Mongolia | 14,571 | 63.80 | 2.90 |
| Ningxia | 10,353 | 34.50 | 3.08 |
| Qinghai | 9069 | 24.30 | 1.82 |
| Shandong | 12,881 | 53.20 | 4.00 |
| Shanghai | 27,001 | 93.30 | 0.42 |
| Shaanxi | 12,126 | 47.10 | 4.64 |
| Shanxi | 10,499 | 36.10 | 3.25 |
| Sichuan | 10,194 | 33.80 | 2.91 |
| Taiwan, China | 32,300 | 96.40 | 0.19 |
| Tianjin | 17,420 | 76.90 | 1.75 |
| Xinjiang | 10,469 | 35.80 | 3.22 |
| Xizang | 9315 | 26.20 | 2.04 |
| Yunnan | 9097 | 24.50 | 1.85 |
| Zhejiang | 17,745 | 78.20 | 1.66 |

WTP: willingness-to-pay threshold, EVPI: expected value of perfect information.

**Supplementary Table S2. Summary of the ICERs for PPSV23 at different prices.**

| Price of PPSV23 ($) | ICER | Price of PPSV23 ($) | ICER |
| --- | --- | --- | --- |
| 0 | −8315.68 | 36 | 18,243.06 |
| 1 | −7577.94 | 37 | 18,980.80 |
| 2 | −6840.19 | 38 | 19,718.54 |
| 3 | −6102.45 | 39 | 20,456.28 |
| 4 | −5364.71 | 40 | 21,194.03 |
| 5 | −4626.97 | 41 | 21,931.77 |
| 6 | −3889.22 | 42 | 22,669.51 |
| 7 | −3151.48 | 43 | 23,407.25 |
| 8 | −2413.74 | 44 | 24,145.00 |
| 9 | −1676.00 | 45 | 24,882.74 |
| 10 | −938.25 | 46 | 25,620.48 |
| 11 | −200.51 | 47 | 26,358.22 |
| 12 | 537.23 | 48 | 27,095.97 |
| 13 | 1274.98 | 49 | 27,833.71 |
| 14 | 2012.72 | 50 | 28,571.45 |
| 15 | 2750.46 | 51 | 29,309.19 |
| 16 | 3488.20 | 52 | 30,046.94 |
| 17 | 4225.95 | 53 | 30,784.68 |
| 18 | 4963.69 | 54 | 31,522.42 |
| 19 | 5701.43 | 55 | 32,260.16 |
| 20 | 6439.17 | 56 | 32,997.91 |
| 21 | 7176.92 | 57 | 33,735.65 |
| 22 | 7914.66 | 58 | 34,473.39 |
| 23 | 8652.40 | 59 | 35,211.14 |
| 24 | 9390.14 | 60 | 35,948.88 |
| 25 | 10,127.89 | 61 | 36,686.62 |
| 26 | 10,865.63 | 62 | 37,424.36 |
| 27 | 11,603.37 | 63 | 38,162.11 |
| 28 | 12,341.11 | 64 | 38,899.85 |
| 29 | 13,078.86 | 65 | 39,637.59 |
| 30 | 13,816.60 | 66 | 40,375.33 |
| 31 | 14,554.34 | 67 | 41,113.08 |
| 32 | 15,292.08 | 68 | 41,850.82 |
| 33 | 16,029.83 | 69 | 42,588.56 |
| 34 | 16,767.57 | 70 | 43,326.30 |
| 35 | 17,505.31 |  |  |

ICER: incremental cost-effectiveness ratio, PPSV23: 23-valent pneumococcal polysaccharide vaccine.

**Supplementary Table S3. Only one dose of PPSV23 at age 50.**

| Strategy | No vaccination | PPSV23 |
| --- | --- | --- |
| Case |  |  |
| Probability of CAP (%) | 37.77 | 37.39 |
| Outpatient pneumonia | 26.71 | 26.44 |
| Inpatient pneumonia | 11.06 | 10.95 |
| Probability of IPD (%) | 0.138 | 0.130 |
| Meningitis | 0.023 | 0.022 |
| Bacteremia | 0.115 | 0.109 |
| Probability of sequelae (%) |  |  |
| Inpatient pneumonia | 0.299 | 0.296 |
| Meningitis | 0.007 | 0.006 |
| Bacteremia | 0.008 | 0.008 |
| Death |  |  |
| CAP | 1.251 | 1.243 |
| IPD |  |  |
| Meningitis | 0.00238 | 0.00231 |
| Bacteremia | 0.01677 | 0.01628 |
| Sequelae | 0.18226 | 0.17884 |
| Cost-Effectiveness |  |  |
| Cost ($) | 264.02 | 280.59 |
| QALYs | 14.86685 | 14.86808 |
| LYs | 20.33848 | 20.34010 |
| Incremental cost | — | 16.57 |
| Incremental QALY | — | 0.00123 |
| Incremental LY | — | 0.00162 |
| Incremental cost per QALY | — | 13,471.12 |
| Incremental cost per LY | — | 10,237.40 |

PPSV23: 23-valent pneumococcal polysaccharide vaccine, CAP: community-acquired pneumonia, IPD: invasive pneumococcal disease, QALY: quality-adjusted life-years, LY: life-year.

**Supplementary Table S4. Characteristics of** **PPSV23 reports submitted to VAERS, United States, January 01 2014-April 26 2024.**

| Report characteristic | <50 years | 50-59 years | 60-69 years | 70-79 years | ≥80 years | All ages^a^ |
| --- | --- | --- | --- | --- | --- | --- |
|  | Total = 4285, n (%) | Total = 2713, n (%) | Total = 8470, n (%) | Total = 5375, n (%) | Total = 1484, n (%) | Total = 29,536, n (%) |
| Sex^b^ |  |  |  |  |  |  |
| Male | 1264 (29.50) | 768 (28.31) | 2312 (27.30) | 1488 (27.68) | 399 (26.89) | 6995 (23.68) |
| Female | 2822 (65.86) | 1889 (69.63) | 5972 (70.51) | 3784 (70.40) | 1049 (70.69) | 17,018 (57.62) |
| Median age^b^ (IQR), years | 34 (22–43) | 55 (52–57) | 66 (65–67) | 73 (71–76) | 83 (81–86) | 66 (55–71) |
| Median TTO^b^ (rang), days | 0 (0–1) | 0 (0–1) | 0 (0–1) | 0 (0–1) | 0 (0–1) | 0 (0–1) |
| PPSV23 given alone | 4171 (97.34) | 2647 (97.57) | 8148 (96.20) | 5200 (96.74) | 1453 (97.91) | 28,807 (97.53) |
| Seriousness |  |  |  |  |  |  |
| Non-serious | 3881 (90.57) | 2549 (93.96) | 8161 (96.35) | 5167 (96.13) | 1374 (92.59) | 28,144 (95.29) |
| Serious, non-death | 395 (9.22) | 160 (5.90) | 304 (3.59) | 202 (3.76) | 97 (6.54) | 1331 (4.51) |
| Serious, death | 9 (0.21) | 4 (0.15) | 5 (0.06) | 6 (0.11) | 13 (0.88) | 61 (0.21) |

^a^ Includes reports missing or unknown age.

^b^ Data missing or unknown for sex (5523, 18.70%), age (7209, 24.41%) and TTO (5022, 17.00%).

PPSV23: 23-valent pneumococcal polysaccharide vaccine, VAERS: vaccine adverse event reporting system, IQR: interquartile range, TTO: time-to-onset.

**Supplementary Table S5. MedDRA PTs with a disproportionality score EBGM05>2.0 grouped by SOC for PPSV23 in VAERS, United States, January 01 2014-April 26 2024.**

| SOC | PT | a | EBGM (EBGM 05) |
| --- | --- | --- | --- |
| Blood and lymphatic system disorders | Leukocytosis | 66 | 2.97 (2.32) |
|  | Bandaemia | 4 | 7.47 (2.57) |
| General disorders and administration site conditions | Injection site erythema | 5892 | 4.08 (3.96) |
|  | Injection site swelling | 5178 | 4.46 (4.33) |
|  | Injection site pain | 5014 | 2.91 (2.82) |
|  | Peripheral swelling | 4009 | 5.61 (5.41) |
|  | Injection site warmth | 2478 | 3.78 (3.62) |
|  | Swelling | 2219 | 4.14 (3.96) |
|  | Injected limb mobility decreased | 1150 | 7.02 (6.59) |
|  | Tenderness | 770 | 3.76 (3.49) |
|  | Injection site reaction | 677 | 3.06 (2.83) |
|  | Injection site induration | 557 | 2.43 (2.23) |
|  | Inflammation | 352 | 2.45 (2.2) |
|  | Induration | 345 | 3.64 (3.26) |
|  | Local reaction | 290 | 5.1 (4.51) |
|  | Injection site inflammation | 285 | 5.06 (4.48) |
|  | Injection site urticaria | 181 | 2.49 (2.14) |
|  | Injection site oedema | 172 | 7.82 (6.64) |
|  | Oedema | 106 | 3.85 (3.15) |
|  | Injection site discomfort | 99 | 2.66 (2.18) |
|  | Injection site streaking | 65 | 12.05 (9.1) |
|  | Injection site irritation | 52 | 3.25 (2.46) |
|  | Injection site hypersensitivity | 37 | 5.05 (3.59) |
|  | Injection site joint pain | 32 | 3.84 (2.67) |
|  | Extensive swelling of vaccinated limb | 26 | 3.66 (2.45) |
|  | Injection site movement impairment | 24 | 4.16 (2.74) |
|  | Administration site swelling | 19 | 7.63 (4.67) |
|  | Injection site ulcer | 9 | 6.72 (3.33) |
|  | Administration site warmth | 6 | 6.07 (2.58) |
|  | Injection site plaque | 6 | 6.07 (2.58) |
|  | Injection site necrosis | 5 | 8.09 (3.1) |
|  | Injection site joint discomfort | 4 | 16.19 (4.87) |
|  | Injection site joint effusion | 3 | 9.71 (2.74) |
|  | Vaccination site necrosis | 3 | 18.21 (4.35) |
| Immune system disorders | Allergy to vaccine | 65 | 2.79 (2.17) |
|  | Type III immune complex mediated reaction | 14 | 5.4 (3.1) |
|  | Serum sickness | 13 | 4.57 (2.58) |
|  | Immunodeficiency common variable | 10 | 22.07 (9.54) |
|  | Hypogammaglobulinaemia | 5 | 10.56 (3.92) |
|  | Selective IgA immunodeficiency | 3 | 18.21 (4.35) |
| Infections and infestations | Cellulitis | 1309 | 10.71 (10.06) |
|  | Injection site cellulitis | 348 | 8.55 (7.61) |
|  | Pneumonia pneumococcal | 183 | 37.03 (27.5) |
|  | Infection | 124 | 2.92 (2.44) |
|  | Pneumococcal infection | 83 | 36.98 (23.8) |
|  | Injection site infection | 76 | 7.27 (5.69) |
|  | Pneumococcal bacteraemia | 54 | 39.73 (21.25) |
|  | Bacterial infection | 36 | 4.05 (2.88) |
|  | Skin infection | 29 | 5.52 (3.75) |
|  | Localised infection | 26 | 4.48 (2.99) |
|  | Meningitis pneumococcal | 19 | 20.06 (11.15) |
|  | Vaccination site cellulitis | 15 | 4.36 (2.57) |
|  | Vaccination site infection | 7 | 4.86 (2.22) |
|  | Pneumonia streptococcal | 5 | 5.92 (2.32) |
|  | Erysipelas | 4 | 10.79 (3.55) |
|  | Septic arthritis streptococcal | 3 | 29.14 (4.87) |
|  | Soft tissue infection | 3 | 8.57 (2.46) |
| Investigations | White blood cell count increased | 319 | 3.83 (3.42) |
|  | Blood culture negative | 82 | 3.21 (2.56) |
|  | Streptococcus test | 72 | 4.4 (3.46) |
|  | Streptococcus test positive | 64 | 7.11 (5.45) |
|  | Antibody test negative | 54 | 3.55 (2.69) |
|  | Neutrophil count increased | 44 | 3.41 (2.51) |
|  | Blood immunoglobulin G decreased | 26 | 10.43 (6.76) |
|  | Blood immunoglobulin A decreased | 17 | 15.88 (8.89) |
|  | Blood immunoglobulin G normal | 17 | 4.13 (2.51) |
|  | Blood immunoglobulin M decreased | 14 | 10.15 (5.63) |
|  | B-lymphocyte count | 8 | 10.5 (4.8) |
|  | CD4 lymphocytes decreased | 7 | 9.19 (4.04) |
|  | Reticulocyte count | 7 | 5.96 (2.7) |
|  | T-lymphocyte count decreased | 7 | 11.72 (5.01) |
|  | Band neutrophil percentage increased | 5 | 9.71 (3.64) |
|  | CD8 lymphocytes decreased | 5 | 18.68 (6.11) |
|  | Corynebacterium test negative | 5 | 26.98 (7.24) |
|  | Arthroscopy | 4 | 6.47 (2.26) |
|  | Corynebacterium test positive | 4 | 12.14 (3.92) |
|  | Natural killer cell count decreased | 4 | 38.85 (4.34) |
|  | Anti-HLA antibody test positive | 3 | 18.21 (4.35) |
|  | CD8 lymphocytes increased | 3 | 36.42 (3.79) |
|  | Peak expiratory flow rate | 3 | 7.28 (2.13) |
| Musculoskeletal and connective tissue disorders | Mobility decreased | 1253 | 3.09 (2.91) |
|  | Musculoskeletal pain | 391 | 4 (3.61) |
|  | Joint range of motion decreased | 210 | 2.45 (2.13) |
|  | Musculoskeletal disorder | 59 | 2.87 (2.21) |
|  | Limb mass | 57 | 3.43 (2.62) |
|  | Muscle swelling | 31 | 3.81 (2.64) |
|  | Muscle oedema | 6 | 6.78 (2.86) |
|  | Fasciitis | 5 | 10.56 (3.92) |
|  | Soft tissue necrosis | 3 | 7.28 (2.13) |
| Skin and subcutaneous tissue disorders | Erythema | 5389 | 5.04 (4.89) |
|  | Skin warm | 2295 | 5.54 (5.29) |
|  | Skin tightness | 76 | 3.8 (3) |
|  | Skin reaction | 74 | 2.74 (2.16) |
|  | Erythema multiforme | 47 | 2.98 (2.22) |
|  | Yellow skin | 14 | 3.98 (2.3) |

PPSV23: 23-valent pneumococcal polysaccharide vaccine, MedDRA: medical dictionary for regulatory activities, PT: preferred term, SOC: system organ class, EBGM: empirical bayesian geometric mean; EBGM05, the lower limit of 95% CI of EBGM, VAERS: vaccine adverse event reporting system.

**Supplementary Figure Captions:**

**Supplementary Figure S1. Model Structure.**

**
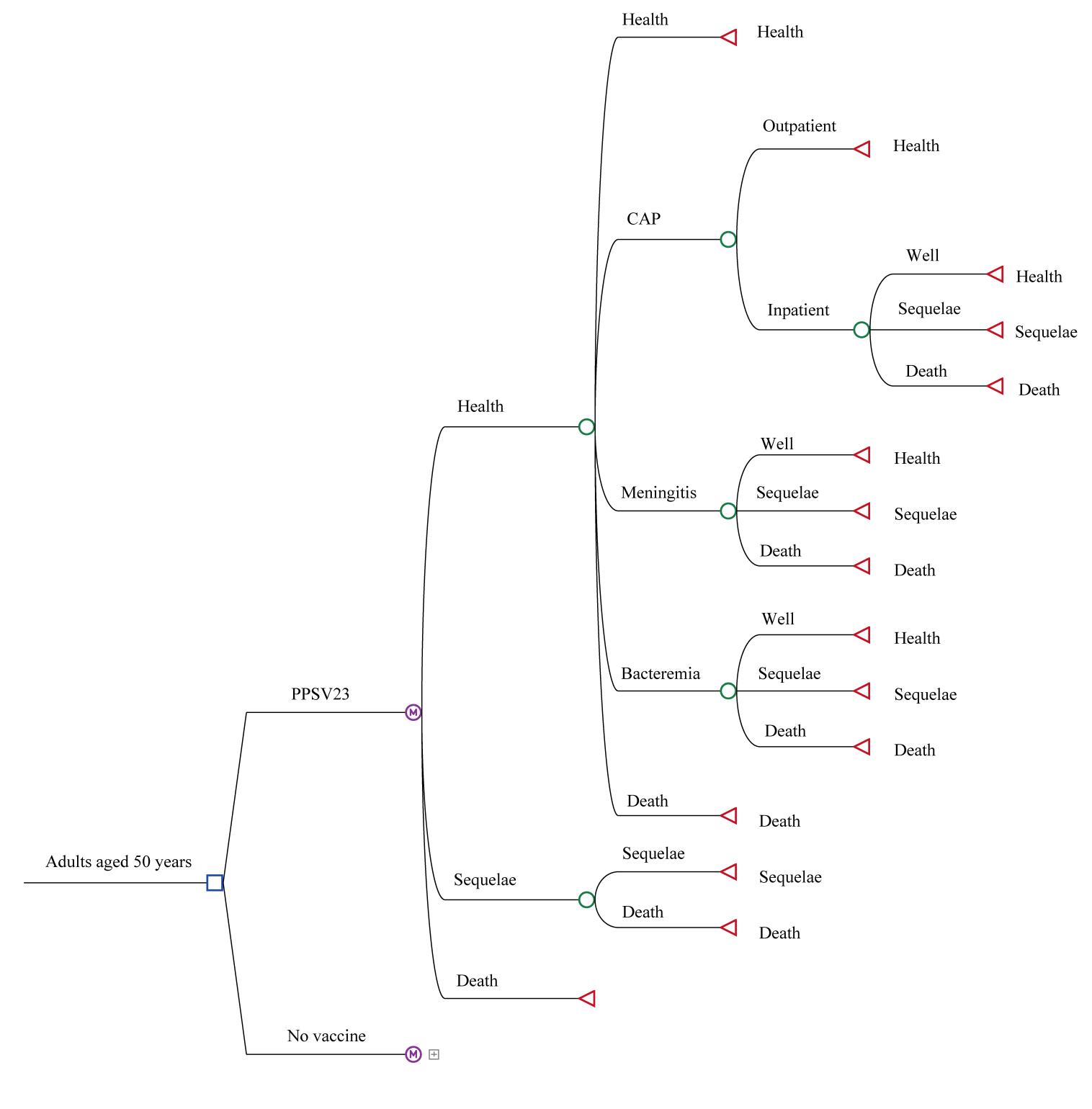
**

**Supplementary Figure S2. The probability of cost-effectiveness for PPSV23 by** **province.**

**
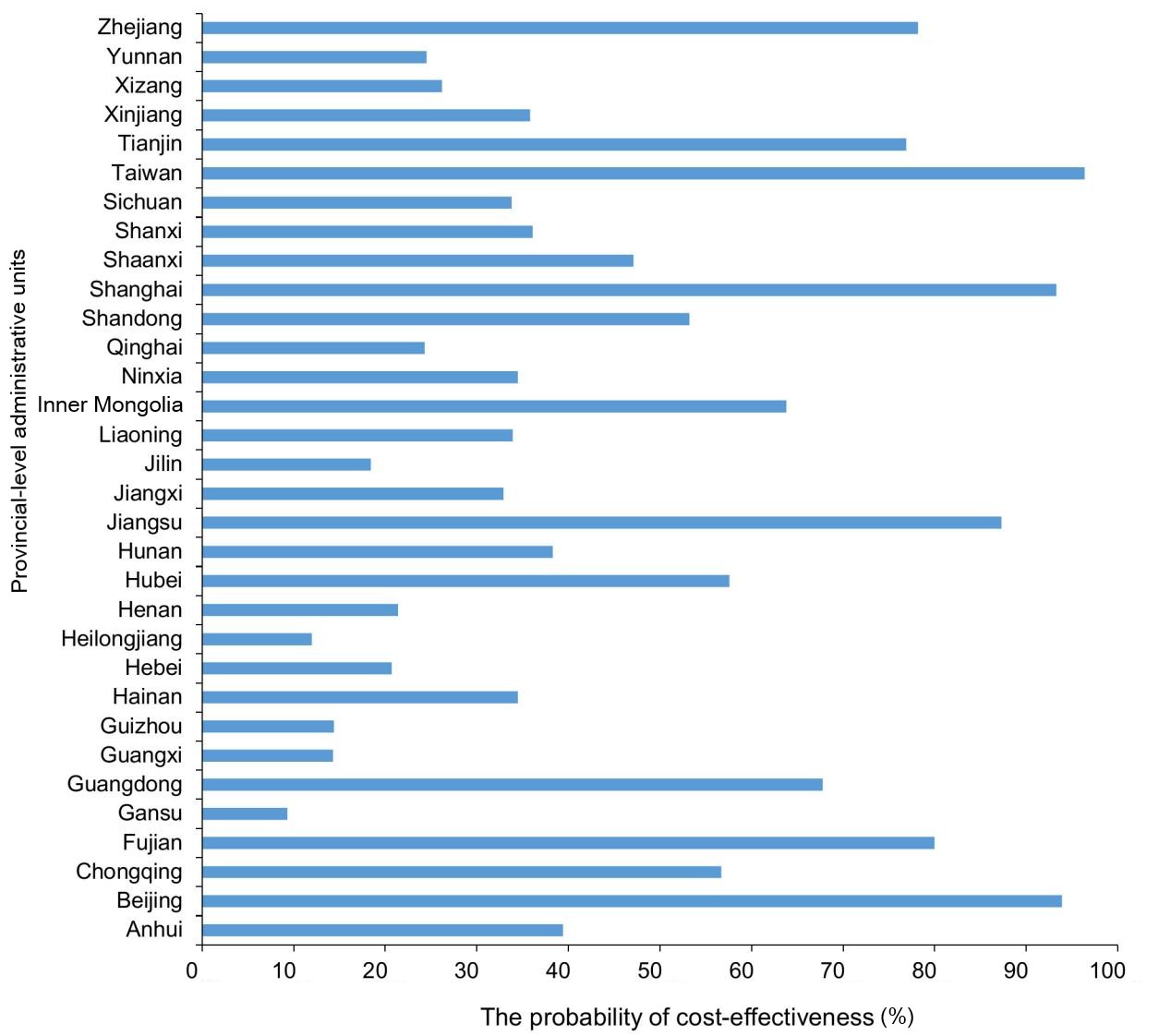
**

**Supplementary Figure S3. Scatter plots for PSA with different prices.**

**
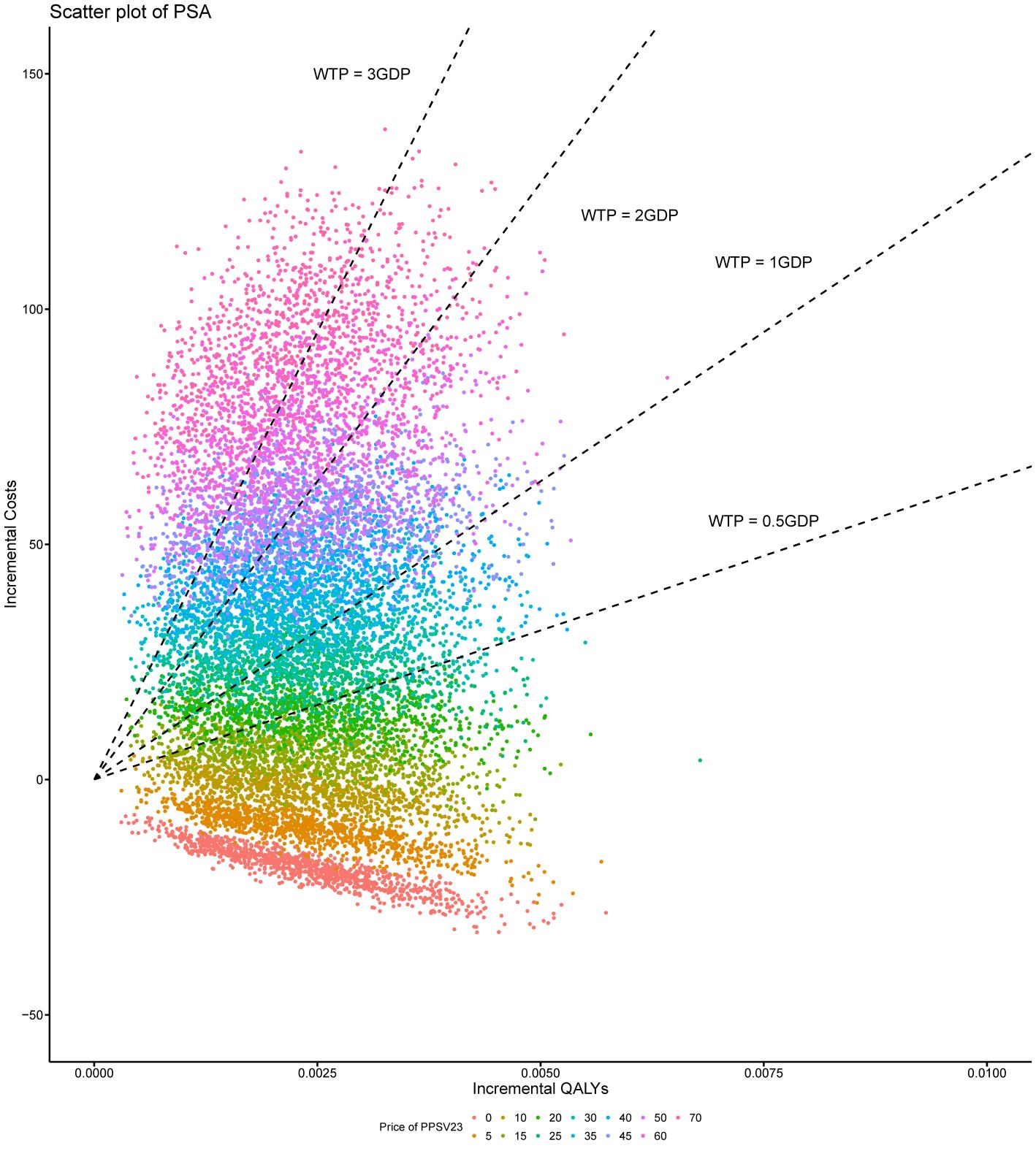
**

**Supplementary Figure S4. VAERS reports from various states.**


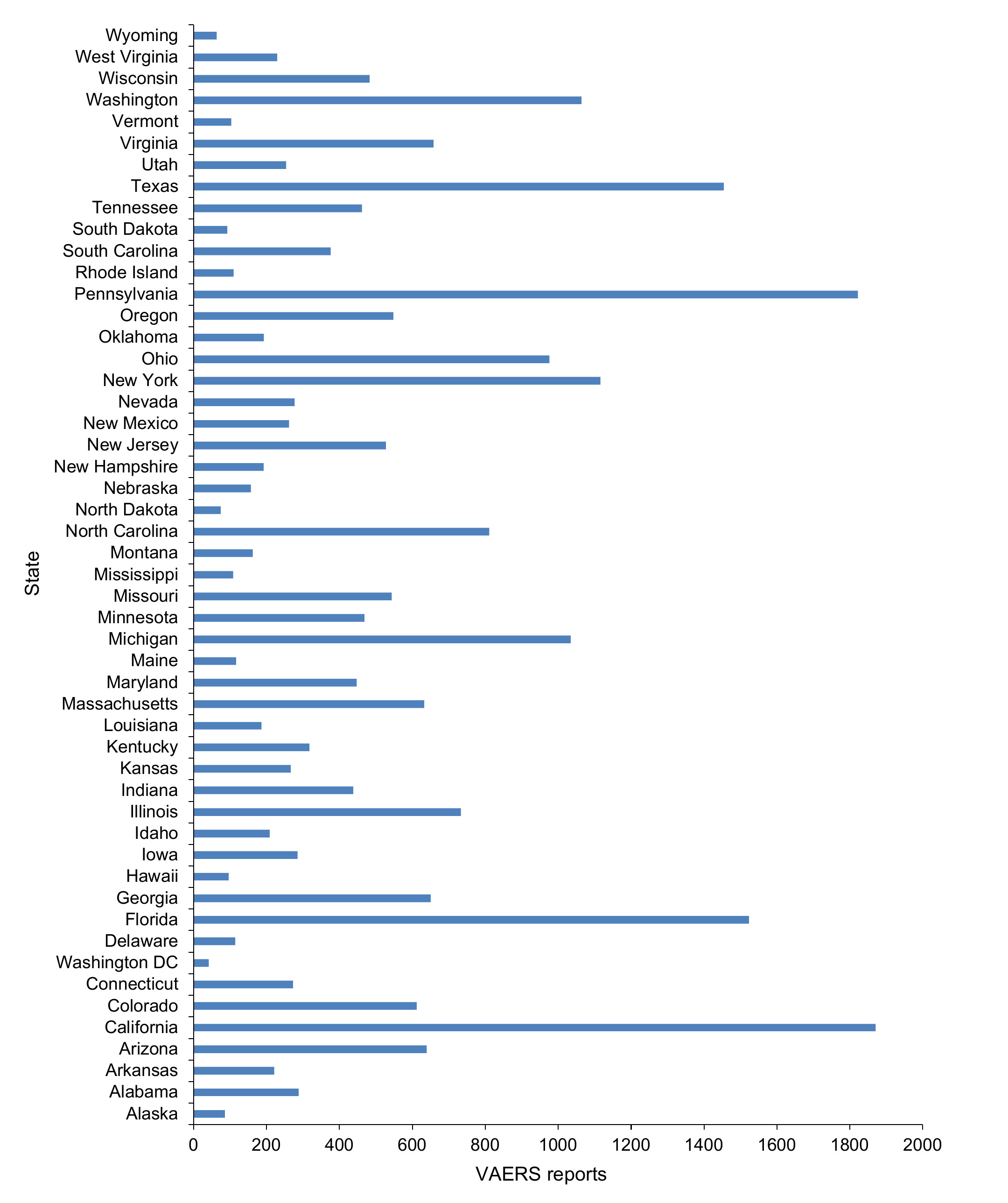

Supplement: Supplementary file 1 [file mmc1.docx]
